# Association between augmented levels of the gut pro-hormone Proneurotensin and subclinical vascular damage

**DOI:** 10.1038/s41598-024-65992-4

**Published:** 2024-07-02

**Authors:** Francesca De Vito, Teresa Vanessa Fiorentino, Antonio Facciolo, Velia Cassano, Maria Resilde Natale, Gaia Chiara Mannino, Elena Succurro, Franco Arturi, Angela Sciacqua, Giorgio Sesti, Francesco Andreozzi

**Affiliations:** 1 Department of Medical and Surgical Sciences, University Magna Graecia of Catanzaro, 88100 Catanzaro, Italy; 2 Department of Clinical and Molecular Medicine, University of Rome-Sapienza, 00189 Rome, Italy

**Keywords:** Proneurotensin, Neurotensin, Cardiovascular disease, Subclinical vascular damage, Carotid intima-media thickness, Pulse pressure, Biomarkers, Cardiology

## Abstract

Elevated levels of the gut pro-hormone Proneurotensin (proNT) have been found to predict development of cardiovascular disease. However, it is still unknown whether higher proNT levels are associated with subclinical vascular damage. Herein, we investigated the relationship between higher proNT concentrations and augmented pulse pressure (PP) and carotid intima-media thickness (cIMT), indicators of increased arterial stiffness and subclinical atherosclerosis, respectively. Clinical characteristics, PP and cIMT were evaluated in 154 non-diabetic individuals stratified into tertiles according to fasting serum proNT concentrations. We found that, subjects with higher proNT levels exhibited a worse lipid profile and insulin sensitivity, increased C-reactive protein levels, along with higher values of PP and cIMT as compared to the lowest proNT tertile. Prevalence of elevated PP (≥ 60 mmHg) and subclinical carotid atherosclerosis (IMT > 0.9 mm) was increased in the highest tertile of proNT. In a logistic regression analysis adjusted for several confounders, subjects with higher proNT levels displayed a fivefold raised risk of having elevated PP values (OR 5.36; 95%CI 1.04–27.28; *P* = 0.05) and early carotid atherosclerosis (OR 4.81; 95%CI 1.39–16.57; *P* = 0.01) as compared to the lowest proNT tertile. In conclusion, higher circulating levels of proNT are a biomarker of subclinical vascular damage independent of other atherosclerotic risk factors.

## Introduction

Cardiovascular diseases (CVDs) represent the leading cause of mortality and a major contributor to disability in the world^1–3^. The Global Burden of Disease Study has estimated that prevalence of CVDs in the last three decades is nearly doubled passing from 271 million cases in 1990 to 523 million in 2019, mainly driven by the growing prevalence of obesity and its related disorders such as hypertension, diabetes and dyslipidemia^1,2^. Several mechanisms not yet completely identified underlie the interplay between obesity and vascular damage including adipose tissue dysfunction, chronic subclinical inflammation, pro-atherogenic lipid profile, disrupted glucose homeostasis, altered intestinal microbioma, and hormonal changes^4,5^.

Recently, rising evidence has demonstrated the role of the intestinal hormone Neurotensin (NT) in the development of obesity and its related cardio-metabolic disorders such as type 2 diabetes, non-alcoholic fatty liver disease (NAFLD), and CVDs^6–12^. NT is a peptide hormone released by the intestinal N cells in response to a fat meal able to regulate gastrointestinal motility and secretion, thus promoting intestinal fatty acid absorption and, consequently, weight gain^6–9^. Additionally, NT has been found to exert pro-inflammatory activities and contribute to adipose tissue dysfunction, ectopic fat accumulation, and insulin resistance^8,10,13–16^. Besides these metabolic effects, NT has a wide range of actions on cardiovascular system including stimulation of cardiac contractility, heart rate acceleration, modulation of vascular tone and permeability^17–23^. The biological actions of NT are mediated by its binding to three receptors, the G-proteins associated receptors NTR1 and NTR2, and NTR3, also called sortilin-1, which is a non–G-protein coupled receptor, known to regulate endocytosis and trafficking of several molecules including cholesterol particles^24^. Genetic or pharmacologic inhibition of NT/NTRs pathway has been found to protect mice from high-fat diet induced weight gain, altered glucose tolerance, hypercholesterolemia, hepatic steatosis, and atherosclerosis^11,12,25,26^. In agreement with these preclinical results, higher circulating concentrations of Proneurotensin (proNT), a stable NT precursor produced in equimolar amounts relative to NT^27^, have been found to be associated with obesity, type 2 diabetes, and NAFLD in humans^9,13,28–32^. Remarkably, prospective studies have demonstrated that augmented fasting proNT levels predict development of CVDs independently of cardio-metabolic risk factors^30,33–35^, and genetic variation in the 1p13 locus containing the NTR3 gene has been linked to an increased risk of coronary artery disease^36^. However, a few studies have assessed cross-sectional association between increased proNT levels and CVDs^37^. Furthermore, since elevated levels of ProNT have been found in subjects with type 2 diabetes, a known pathogenic factor of vascular damage, whether the association between higher proNT and CVDs is independent of diabetes status needs to be ascertain. Moreover, it is currently unknown whether raised circulating levels of proNT are associated with subclinical vascular damage. To address this issue, we aimed to explore the relationship between circulating levels of proNT and carotid intima-media thickness (IMT), an indicator of early atherosclerosis, and pulse pressure, a surrogate measure of arterial stiffness, both known predictors of adverse cerebrocardiovascular outcomes^38–41^, in a well-characterized cohort of non-diabetic subjects.

## Results

The study group encompassing 154 individuals with a mean age of 45.7 ± 11.7 years, and a mean BMI of 31.3 ± 5.9 kg/m^2^, was subdivided into tertiles according to the fasting serum concentrations of proNT. Demographic, anthropometric and biochemical characteristics of individuals in the lowest, intermediate and highest tertiles of proNT are summarized in Table 1. No significant difference regarding age, sex, anthropometric measures including BMI and waist circumference, smoking habit, glucose tolerance status, and family history of CVDs were observed between the three study groups. We found that subjects within the highest tertile of proNT exhibited a worse cardio-metabolic risk profile having increased levels of triglycerides, 2 h post-load insulin, hsCRP, and lower values of HDL cholesterol and whole body insulin sensitivity estimated by Matsuda index as compared to the lowest proNT group (Table 1).

**Table 1:** Anthropometric and metabolic characteristics of study participants stratified on the basis of fasting proNT levels.

	1 Tertile (n = 51)	2 Tertile (n = 51)	3 Tertile (n = 52)	*P*
Sex (M/F)	20/31	29/22	27/25	0.184
Age (years)	47 ± 11	44 ± 10	45 ± 13	0.331
Positive family history for cardiovascular disease n. (%)	13 (25%)	9 (18%)	12 (23%)	0.524
Smoking habit (Current/Never/Ex-smokers)	32/9/10	32/11/8	29/11/12	0.872
BMI (kg/m^2^)	30.3 ± 5.2	32.1 ± 6.1	31.1 ± 6.4	0.160
Waist circumference (cm)	100 ± 14	105 ± 15	105 ± 16	0.177
Systolic blood pressure (mmHg)	129 ± 13	130 ± 17	128 ± 13	0.669
Diastolic blood pressure (mmHg)	84 ± 9	84 ± 11	77 ± 11*** ^###^	0.001
Heart rate (bpm)	71 ± 10	71 ± 9	70 ± 7	0.643
Total cholesterol (mg/dl)	207 ± 31	194 ± 39	206 ± 46	0.215
HDL-C (mg/dl)	53 ± 15	48 ± 12*	47 ± 11**	0.03
Triglycerides (mg/dl)	109 ± 62	123 ± 50	136 ± 89*	0.074
Fasting glucose (mg/dl)	94 ± 10	94 ± 9	91 ± 11	0.140
2 h post-load glucose (mg/dl)	116 ± 25	118 ± 22	119 ± 29	0.801
Fasting insulin (mU/ml)	14 ± 8	16 ± 8	15 ± 9	0.297
2 h post-load insulin (mU/ml)	86 ± 75	92 ± 60	117 ± 106*	0.138
NGT/IFG/IGT/combo IFG-IGT	35/7/5/4	37/7/2/5	36/7/7/2	0.67
Matsuda index	58 ± 64.5	38.4 ± 36.8*	19.5 ± 30.7***	0.001
hsCRP (mg/dl)	2.3 ± 1.7	4.0 ± 3.2**	4.5 ± 3.8*	0.022
ProNT (pg/ml)	6 ± 4	51 ± 36***	304 ± 106*** ^###^	< 0.0001
Pulse pressure (mmHg)	45 ± 9	47 ± 12	51 ± 13* ^#^	0.05
Mean Carotid IMT (mm)	0.7 ± 0.2	0.8 ± 0.2*	0.9 ± 0.2***	0.001
Maximum carotid IMT (mm)	0.8 ± 0.2	0.9 ± 0.2	1.0 ± 0.2**	0.01

Data are means ± SD. Fasting and 2 h post-load insulin, proNT, triglycerides, HDL-C and hsCRP were log transformed for statistical analysis, but values in the table represent back transformation to the original scale. Categorical variables were compared by χ^2^ test. Comparisons between the three groups were performed using a general linear model.

*BMI* body mass index, *HDL-C* high-density lipoprotein-cholesterol, *carotid IMT* carotid intima-media thickness, *hsCRP* high-sensitivity C-reactive protein, *ProNT* proneurotensin, *NGT* normal glucose tolerance, *IFG* impaired fasting glucose, *IGT* impaired glucose tolerance.

*P < 0.05 versus 1st proNT tertile; **P < 0.01 versus 1st proNT tertile; ***P < 0.0001 versus 1st proNT tertile.

^#^*P* < 0.05 versus 2nd proNT tertile; ^###^*P* < 0.0001 versus 2nd proNT tertile.

**Figure 1:**
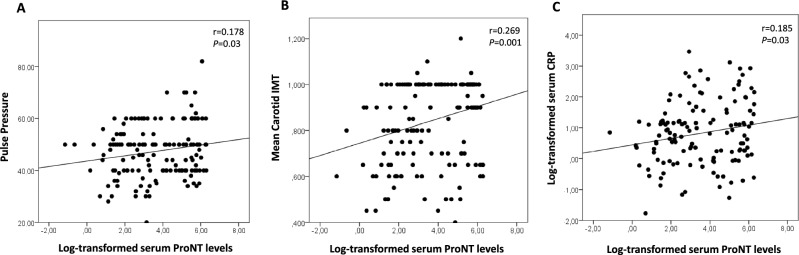
Univariate analysis between fasting serum levels of pro-NT (naturally log transformed) and pulse pressure (**A**), mean carotid IMT (**B**) and serum CRP levels (**C**).

**Figure 2:**
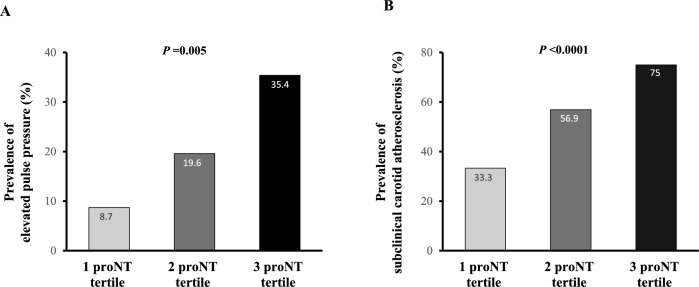
Prevalence of elevated pulse pressure (**A**) and early carotid atherosclerosis (**B**) amongst study subjects subdivided into tertiles according to fasting serum proNT levels.

Higher proNT levels were associated with subclinical signs of vascular damage, with subjects in the highest tertile of proNT exhibiting increased levels of pulse pressure and mean carotid IMT as compared to the lowest proNT tertile (Table 1). We also found that maximum carotid IMT values were significantly increased in subjects with higher levels of proNT as compared to lowest proNT tertile (Table 1). Moreover, by performing univariate analysis we found a positive association between serum proNT levels and pulse pressure, carotid IMT, and serum CRP values (Fig. 1A–C). Accordingly, proportion of subjects having elevated pulse pressure (≥ 60 mmHg) (Fig. 2A), and subclinical carotid atherosclerosis (mean carotid IMT > 0.9 mm) was significantly higher in the highest tertile of proNT as compared to the lowest tertile (Fig. 2B).

**Figure 3:**
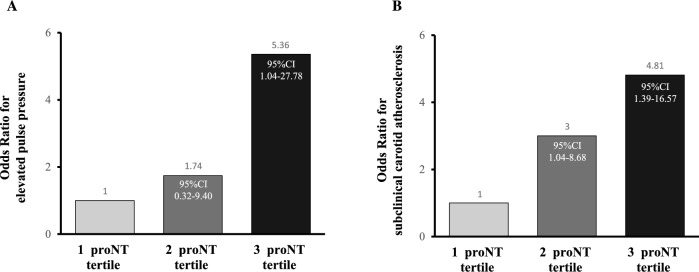
Logistic regression analyses of the association between the tertiles of proNT levels and elevated pulse pressure (**A**) and early carotid atherosclerosis (**B**).

Next, we carried out logistic regression analyses to investigate the independent relationship between higher circulating proNT levels and presence of subclinical vascular damage. In a logistic regression model adjusted for several cardio-metabolic risk factors including age, sex, BMI, triglycerides, total and HDL cholesterol, hsCRP and Matsuda index of insulin sensitivity, we found that, as compared to individuals in the lowest proNT tertile, those in the highest tertile of proNT exhibited a fivefold increased risk of having elevated pulse pressure (≥ 60 mmHg) (OR 5.36; 95%CI 1.04–27.28; *P* = 0.047); whereas a not significant 1.7-fold increased risk was observed in subjects in the intermediate proNT tertile (OR 1.74; 95%CI 0.32–9.40; *P* = 0.5) (Fig. 3A).

Moreover, in a logistic regression model adjusted for age, sex, BMI, SBP, DBP, triglycerides, total and HDL cholesterol, hsCRP and Matsuda index, individuals in the intermediate and highest tertiles of proNT showed a threefold (OR 3.00; 95%CI 1.04–8.68; *P* = 0.04) and 4.8-fold (OR 4.81; 5%CI 1.39–16.57; *P* = 0.01) higher risk of having subclinical carotid atherosclerosis (mean carotid IMT > 0.9 mm) in comparison to those with lower levels of proNT, respectively (Fig. 3B).

The independent association between higher proNT and subclinical vascular damage was retained even after excluding subjects with altered glucose tolerance (IFG and/or IGT) (n = 44). Indeed, we found that amongst subjects with NGT (n = 110) those in the highest tertile of proNT exhibited a sevenfold increased risk of having elevated pulse pressure (≥ 60 mmHg) (OR 7.16; 95%CI 1.10–47.12; *P* = 0.04), and a fourfold higher risk of having subclinical carotid atherosclerosis (carotid IMT > 0.9 mm) (OR 3.74, 95CI 1.03–13.61, *P* = 0.04) in comparison to those with lower levels of proNT.

In an attempt to identify the determinants of serum levels of proNT we built a linear regression model including age, sex, BMI, SBP, DBP, triglycerides, total and HDL cholesterol, hsCRP and Matsuda index. We found that triglycerides (β = 0.23, *P* = 0.03) and insulin sensitivity assessed by Matsuda index (β = − 0.26, *P* = 0.01) were independent contributors of proNT levels.

## Discussion

Early identification of subjects at increased risk of CVDs is crucial for applying preventive approaches in order to reduce adverse clinical outcomes. This need has prompted several efforts to identify novel circulating biomarkers able to improve cardiovascular risk assessment beyond the traditional risk factors^42^. The identification of circulating biomarkers of vascular damage has not only an undoubted clinical utility as a diagnostic tool but also might provide new insights into the complex pathophysiology of CVDs.

Prior longitudinal studies have demonstrated that increased levels of proNT, the stable precursor of the gut hormone NT, are associated with an increased risk of developing cardiovascular diseases independently of traditional cardiovascular risk factors^30,33–35^. This evidence coupled with those obtained from preclinical studies in animal models showing that inhibition of NT/NTR pathway reduced atherosclerosis, plaque area, vascular inflammation and calcification^11,26,43,44^, suggest that elevated circulating NT levels not only are a biomarker of cardiovascular risk but may also contribute to the pathogenesis of cardiovascular organ damage. In the present investigation we report, for the first time, that amongst non-diabetic individuals those having higher circulating levels of proNT displayed a worse cardio-metabolic risk profile showing increased levels of triglycerides, hsCRP, lower values of HDL cholesterol and insulin sensitivity estimated by the Matsuda index, along with higher values of mean carotid IMT and pulse pressure, two well-established early surrogate markers of vascular remodeling and predictors of cardiovascular events^38–41^. Additionally, we report that higher levels of proNT were associated with increased maximum carotid IMT values, known to have a better predictive value for future cardiovascular events^45,46^. Notably, by performing logistic regression analyses we found that increased levels of proNT were associated with elevated arterial stiffness, defined as pulse pressure ≥ 60 mmHg, and early carotid atherosclerosis, defined as carotid IMT > 0.9 mm, independently of the traditional and risk-enhancers cardiovascular risk factors including age, sex, BMI, lipid profile, blood pressure (only for carotid IMT), hsCRP and insulin sensitivity.

Overall our results showing for the first time the association between higher levels of ProNT and subclinical vascular damage independent of several cardio-metabolic risk factors highlight the pathogenic role of upregulated NT/NTR signaling in the development of atherosclerotic lesions and the consequent CVDs, and, on the other hand, support the clinical utility of measurement of serum proNT levels for a better cardiovascular risk assessment.

Several mechanisms may explain the link between increased proNT levels and subclinical vascular damage. It has been previously shown that NT plays a pivotal role in the pathogenesis of obesity and its metabolic disarrangements including alterations of glucose homeostasis^9–11,28–30^. We observed that the three study groups of proNT levels did not differ for anthropometric measures such as BMI and waist circumference, and glucose tolerance status thus arguing against the possibility that increased fat accumulation and glucose intolerance mediate the effect of NT on vascular damage. In agreement with prior studies^9,13,31^, we observed an association between higher proNT levels and a worse lipid profile and insulin sensitivity estimated by Matsuda index^4,5,47^. However, in a logistic regression model including these potential confounders, we found that subjects in the highest tertile of proNT levels displayed a significantly increased risk of having subclinical vascular damage as compared to those with lower levels of proNT, suggesting that higher proNT levels confers an increased risk of vascular damage regardless lipid profile and whole body insulin responsiveness. Chronic subclinical inflammation is another potential factor implicated in the association between higher proNT levels and cardiovascular organ damage. In line with prior studies showing the pro-inflammatory properties of NT, we observed that subjects with higher proNT levels displayed increased levels of the pro-inflammatory marker hsCRP, a broadly recognized predictor of CVDs^48^. However, we found that the association between higher levels of proNT and vascular damage was independent of hsCRP values, but given the complexity and redundancy of inflammatory pathways involved in the development of atherosclerosis^49^, the role of other pro-inflammatory factors in mediating the negative effect of proNT on vascular biology may not be firmly excluded. Indeed, NT has been found to stimulate the release of several cytokines such as IL-1, IL-6, and IL-8 by activating the pro-inflammatory extracellular signal–regulated kinases (ERK), nuclear factor-kB (NF-kB) and protein kinase C δ (PKCδ) pathways^14–16^, which play a crucial role in vascular dysfunction and remodeling^49,50^. A number of experimental studies have indicated that NT may influence cardiovascular system by indirect mechanisms involving central neuronal circuits, renin–angiotensin–aldosterone system, the release of catecolamines and histamines^17,20,22,51,52^, and by direct interaction with its receptors found to be expressed in endothelial and vascular smooth muscle cells, resulting in increased intracellular calcium release, vascular calcification, oxidative stress and altered trafficking of lipoproteins^21,44,53,54^. However, deeper investigations are warranted to better elucidate the mechanisms by which augmented circulating NT levels may impact functional and structural vascular integrity, and contribute to the pathogenesis of atherosclerosis.

The present study has several strengths such as the inclusion of both men and women, a comprehensive clinical characterization including evaluation of glucose homeostasis by OGTT, the centralization of biochemical assays, the exclusion of conditions known to affect lipid, glucose and cardiovascular homeostasis, and the ultrasound determination of carotid IMT carried out by an experienced operator who was blinded to the clinical data of the participants.

Nevertheless, a number of limitations needs to be acknowledged. First, sample size was relatively small even though power calculation allows us to exclude type I error. Second, we enrolled only White adult subjects, therefore we cannot establish whether our findings may be exploited to other ethnic groups. Additionally, arterial stiffness was estimated by evaluation of peripheral pulse pressure rather than the gold standard noninvasive measure pulse wave velocity. However, pulse pressure is a well-established proxy of arterial stiffness both in clinical setting and epidemiological studies, and has been shown to be a good predictor of adverse cardiovascular events. Furthermore, given the cross-sectional design of the study, our data do not prove a causal relationship between higher NT levels and vascular damage. Moreover, we measured levels of proNT in serum samples collected after an overnight fasting, and whether increased fasting levels of proNT reflect an augmented secretion of NT after a meal needs to be confirmed. Finally, we cannot exclude the effect of uninvestigated potential confounders, including dietary intake of lipid known to promote NT secretion and atherosclerosis, and genetic factors affecting NTR expression/function and lipoprotein metabolism.

In conclusion, our data provide evidence that augmented circulating levels of proNT may confer an increased risk of having subclinical vascular damage in non-diabetic subjects independently of other cardio-metabolic risk factors. The present findings, in line with prior experimental evidence, suggest that the up-regulated NT axis is involved in the pathogenesis of vascular damage and may contribute to the development of atherosclerotic lesions thus becoming a potential target for the treatment of CVDs.

## Methods

The study sample included 154 non-diabetic subjects participating to the CATAnzaro MEtabolic RIsk factors (CATAMERI) study, an ongoing longitudinal observational investigation recruiting adult White individuals carrying at least a cardio-metabolic risk factor, whose protocol details have been previously described^32,55^. All subjects were consecutively recruited from January 2012 to January 2017 at the Department of Medical and Surgical Science of the University of “Magna Graecia” of Catanzaro. We excluded from the present investigation individuals with diabetes mellitus defined according to the current ADA criteria^56^, history of CVDs, autoimmune or malignant affections, chronic gastrointestinal disorders associated with malabsorption, undergone to bariatric surgery, history of alcohol or drug abuse, positivity for antibodies to hepatitis C virus or hepatitis B surface antigen, liver or kidney failure, taking antihypertensive medications or drugs known to affect lipid and glucose homeostasis including statins, estroprogestins and glucocorticoids.

All subjects underwent to a detailed family and personal medical history, and physical examination with collection of anthropometric parameters including body mass index (BMI) and waist circumference. Smoking habit was assessed by self-reported questionnaire and study participants were classified as never smokers, current smokers or ex-smokers. Readings of clinical blood pressure were carried out after a 5 min rest at the right upper arm in the sitting position with a sphygmomanometer for three times and the average was computed. Heart rate was determined electrocardiographically after at least 30 min of quiet rest.

After an overnight fasting, each study participants underwent to biochemical characterization including a 75 g oral glucose tolerance test (OGTT) with basal, 30, 60, 90, and 120 min sampling for plasma glucose and insulin determination. On the basis of OGTT data, individuals were categorized as having normal glucose tolerance (NGT) when fasting plasma glucose was < 100 mg/dl and 2 h post-load glucose < 140 mg/dl, isolated impaired fasting glucose (IFG) when fasting plasma glucose was 100–125 mg/dl and 2 h post-load glucose < 140 mg/dl, and impaired glucose tolerance (IGT) when fasting plasma glucose was < 100 mg/dl and 2 h post-load glucose was 140–199 mg/dl^56^.

The study protocol was conformed to the principles outlined in the Helsinki Declaration and was authorized by the Hospital ethical committee (Comitato Etico Azienda Ospedaliera “Mater Domini”). All study participants gave their written informed consent before to be enrolled in this investigation.

### Ultrasound measurement of carotid IMT

High-resolution B-mode ultrasound scan was carried out in each study participant by a trained examiner blinded to the patients’ clinical features by using an ATL HDI 3000 ultrasound system (Advanced Technology Laboratories, Bothell, WA) equipped with a 5-MHz linear array mechanical transducer^55^. Common carotid artery was scanned and IMT measurement were computed in plaque-free portions of the 10-mm linear segment proximal to the beginning of the dilatation of the carotid bulb. For each individual, two measurements were performed bilaterally and the values were averaged to obtain the mean value of carotid IMT. The maximum IMT was defined as the greatest IMT value measured in either the right or left common carotid artery scanned area^46^.

### Proneurotensin ELISA assay

Serum samples were collected from each overnight-fasted participant, immediately frozen after separation, and stored at − 80 °C until use. An enzyme-linked immunosorbent assay kit was used to determine serum proNT concentrations according to manufacturers’ instructions (MyBioSource, San Diego, CA, USA). Luminescent signals were detected with a plate reader luminometer (Varioskan LUX Multimode Microplate Reader, Thermo Fisher Scientific, Waltham, MA USA, USA). The analytical assay sensitivity was less than 4.78 pg/mL. Intra- and interassay coefficients of variability were less than 10% and 12%, respectively.

Study participants were stratified into tertiles according to the circulating fasting proNT levels.

### Biochemical parameters

Glucose, triglycerides, total and HDL cholesterol levels were assayed by enzymatic methods (Roche, Basel, Switzerland). Serum insulin concentrations were measured by a chemiluminescence-based assay (Immunolite, Siemens, Italy). Levels of serum high sensitivity C reactive protein (hsCRP) were determined using an automated instrument (Cardio-Phase hsCRP, Milan, Italy).

### Calculation

Pulse pressure was calculated as the difference between systolic blood pressure (SBP) and diastolic blood pressure (DBP). A value of pulse pressure ≥ 60 mmHg was used as an indicator of increased vascular stiffness^57^.

A value of IMT ≥ 0.9 mm was considered as indicator of early vascular atherosclerosis^57^.

Whole body insulin sensitivity was estimated by the Matsuda index calculated as follow: 10,000/square root of [fasting glucose x fasting insulin] × [mean glucose × mean insulin during OGTT]^58^.

### Statistical analysis

Variables with skewed distribution including proNT, triglycerides, fasting, and 2 h post-load insulin were natural log-transformed for statistical analyses. Continuous parameters are reported as mean ± SD. Categorical variables were compared by χ2 test. Differences in biochemical and clinical parameters among the three study groups of proNT levels were tested by a general linear model with post hoc Fisher's least significant difference correction for pairwise comparisons. A logistic regression analysis model including several cardio-metabolic confounders was built to estimate the association between the proNT tertiles and risk of subclinical vascular damage defined as increased values of mean carotid IMT (> 0.9 mm) or pulse pressure (≥ 60 mmHg). We evaluated Pearson correlation coefficients to test the association between serum proNT levels and cardio-metabolic parameters. A multiple linear regression analysis was performed to assess the independent contribution of cardio-metabolic parameters to proNT concentrations.

We used SPSS software program Version 22.0 for Windows to carried out the statistical analyses described above. A *p* value < 0.05 was considered statistically significant.

Given the lack of previous studies exploring the relationship between proNT levels and subclinical vascular damage, precluding us to a priori calculate the required sample size for the present investigation, we performed a post hoc power calculation by using G power 3.1. program. Considering the means and SD of carotid IMT detected in the three tertiles of proNT, we found that a sample size of 153 subjects (51 individuals for each study groups) conferred to the study a power of 85% to detect 15–20% of difference in IMT values between the study groups with a level of significance of 5%.

## Data Availability

Data are available upon reasonable request to the corresponding author.
